# Accuracy of Two Motor Assessments during the First Year of Life in Preterm Infants for Predicting Motor Outcome at Preschool Age

**DOI:** 10.1371/journal.pone.0125854

**Published:** 2015-05-13

**Authors:** Alicia J. Spittle, Katherine J. Lee, Megan Spencer-Smith, Lucy E. Lorefice, Peter J. Anderson, Lex W. Doyle

**Affiliations:** 1 Murdoch Childrens Research Institute, Melbourne, Australia; 2 University of Melbourne, Melbourne, Australia; 3 Royal Women’s Hospital, Melbourne, Australia; 4 Monash University, Melbourne, Australia; 5 Royal Children’s Hospital, Melbourne, Australia; The Research Institute at Nationwide Children's Hospital, UNITED STATES

## Abstract

**Aim:**

The primary aim of this study was to investigate the accuracy of the Alberta Infant Motor Scale (AIMS) and Neuro-Sensory Motor Developmental Assessment (NSMDA) over the first year of life for predicting motor impairment at 4 years in preterm children. The secondary aims were to assess the predictive value of serial assessments over the first year and when using a combination of these two assessment tools in follow-up.

**Method:**

Children born <30 weeks’ gestation were prospectively recruited and assessed at 4, 8 and 12 months’ corrected age using the AIMS and NSMDA. At 4 years’ corrected age children were assessed for cerebral palsy (CP) and motor impairment using the Movement Assessment Battery for Children 2^nd^-edition (MABC-2). We calculated accuracy of the AIMS and NSMDA for predicting CP and MABC-2 scores ≤15^th^ (at-risk of motor difficulty) and ≤5^th^ centile (significant motor difficulty) for each test (AIMS and NSMDA) at 4, 8 and 12 months, for delay on one, two or all three of the time points over the first year, and finally for delay on both tests at each time point.

**Results:**

Accuracy for predicting motor impairment was good for each test at each age, although false positives were common. Motor impairment on the MABC-2 (scores ≤5^th^ and ≤15^th^) was most accurately predicted by the AIMS at 4 months, whereas CP was most accurately predicted by the NSMDA at 12 months. In regards to serial assessments, the likelihood ratio for motor impairment increased with the number of delayed assessments. When combining both the NSMDA and AIMS the best accuracy was achieved at 4 months, although results were similar at 8 and 12 months.

**Interpretation:**

Motor development during the first year of life in preterm infants assessed with the AIMS and NSMDA is predictive of later motor impairment at preschool age. However, false positives are common and therefore it is beneficial to follow-up children at high risk of motor impairment at more than one time point, or to use a combination of assessment tools.

**Trial Registration:**

ACTR.org.au ACTRN12606000252516

## Introduction

Very preterm infants (born at <32 weeks’ gestational age) have delayed motor development trajectories during the first year of life compared with infants born at term.[1] The development of movement is a multifaceted process that begins within the first trimester of pregnancy and is influenced by not only personal characteristics but also the environment.[2,3] Several studies have demonstrated that very preterm infants, as a group, perform below term-born peers on standardised tests of motor development throughout the first year of life, and are slower to attain motor skills such as rolling, sitting, crawling, standing and walking.[1, 4–7] Throughout the first year of life, very preterm infants might exhibit extensor postures due to an imbalance between flexor and extensor muscles, transient dystonia, lack of fluency with movement and postural instability due to alterations in the developing central nervous and/or musculoskeletal system.[2–5] Motor difficulties in very preterm infants can persist beyond the first year of life, with childhood motor impairment one of the most commonly reported adverse outcomes of being born preterm.[3] The prevalence of cerebral palsy (CP) increases with decreasing gestational age and a recent systematic review reported the rate of CP at approximately 15% for children born <28 weeks’ gestation, and 6% for those born between 28–31 weeks’ gestation.[8,9] Also of great concern are deficits in gross and fine motor control, balance and coordination in preterm children without CP, which are more common than CP, with children born <32 weeks’ or <1500 g six times more likely to have a moderate motor impairment, and nine times more likely to have a mild impairment than children born at term.[10] Although these motor impairments are often considered to be mild in comparison with CP, their impacts are far-reaching and can influence learning, attention and self-esteem.[3]

It has been recommended that very preterm children have a structured, age-appropriate neuromotor examination at least twice during the first year of life to identify those at risk of future motor difficulties.[11] Whilst the early detection of true motor impairment is important for timely intervention, motor delay diagnosed in the first year might only be transient and disappear as the central nervous system matures.[1,12] It is essential that tools to assess motor development used in clinical follow-up are sensitive enough to detect motor problems ranging from mild impairments through to CP, but also have good specificity, so that infants are not being over-diagnosed with motor delay.[13]

Several tools to assess early motor development are available, which vary in clinical utility (e.g. length of administration, training requirements, and handling of the infant) and psychometric properties (e.g. predictive validity and reliability). Several systematic reviews have demonstrated that the General Movements (GMs) assessment and Test of Infant Motor Performance (TIMP) are useful in early infancy for predicting later outcome, however, they cannot be used after 4–5 months of age.[13,14] The Alberta Infant Motor Scale (AIMS) and NSMDA (Neuro-Sensory Motor Development Assessment) have also been reported to have good psychometric properties and clinical utility when used from 4 months of age compared with other assessment tools available.[13] However, whilst both tools are predictive of motor impairment during infancy,[13,15] there is less known about their long-term predictive validity. It is essential that the longterm predictive validity of the early motor assessments for very preterm children is examined given it is unclear whether gross motor delay in very preterm infants is a variant of typical gross motor development or it reflects long-term impairment.[1]

The primary aim of this study was to evaluate the accuracy of the AIMS and NSMDA during the first year of life for identifying motor impairment in very preterm children at 4 years. The secondary aim was to examine whether the accuracy of the AIMS and NSMDA improved with repeated assessments over the first year of life. Due to the wide natural variation in motor development seen during the first year of life, several authors have suggested that assessments at multiple time points should be used to guide clinical decisions, rather than making a decision based on a single assessment.[16–18] Nonetheless, there are few studies that have examined the accuracy of early motor assessments when used at multiple time points. Further, we assessed the accuracy of combining the assessment findings at multiple time points.

## Methods

### Participants

The study cohort was recruited as part of a previously published randomised controlled trial of a preventive care program to improve developmental outcomes, which recruited 120 very preterm infants from the Royal Women’s Hospital or Royal Children’s Hospital, Melbourne, Australia.[19] The first 99 children randomised to the study, between January 2005 and September 2006, participated in a study of serial motor assessments over the first year of life. To be included in the trial infants had to be born at <30 weeks’ gestation and have a parent who could speak English. Children were excluded if they had a congenital abnormality or lived further than 100 km from the Royal Women’s Hospital. There was little evidence of differences in motor performance between children in the intervention and control groups at 4 years’ corrected age, therefore the data for both groups were pooled in the current study.[20] Ethics approval for the trial and the serial motor assessments study was obtained from the Royal Women’s Hospital and Royal Children’s Hospital, Melbourne Australia. Parents provided written informed consent for their child to participate in the assessments in infancy and separately at 4 years’ corrected age. Perinatal data were recorded during the neonatal period by a research nurse including birth weight, gestational age at birth, postnatal corticosteroids, and oxygen use at 36 weeks’ corrected age.

### Predictor measures

#### Assessments during the first year of life

Infants were assessed at 4, 8 and 12 months’ corrected age at home or the Royal Children’s Hospital, using the AIMS and the NSMDA by one of six physiotherapists or occupational therapists who had attended a training workshop with an author of each of the assessment tools. Reliability between assessors was established prior to the study commencement and all assessors were blinded to the infant’s clinical history.

#### Alberta Infant Motor Scale (AIMS)

The AIMS was designed to monitor motor development in infants at risk of central nervous system dysfunction, such as preterm infants, who might display subtle deviations in performance that other motor assessment tools might not be sensitive to detect.[21] It can be used from birth through to 18 months of age or when the infant begins to walk, and involves observing the infant in prone, supine, sitting and standing with minimal handling. The published normative data for the AIMS are based on a sample of 2,202 infants from Alberta, Canada. A recent cross-sectional study of 650 infants from Canada has demonstrated no change in the sequence and age of AIMS items 20 years on from the original test being developed.[22] The AIMS is appealing to researchers and clinicians due to its ease in administration and strong psychometric properties.[13,21] While the AIMS was not primarily designed as a diagnostic assessment, the predictive validity for later motor outcome in infancy has been examined in several studies.[13] Cut-offs at the 10^th^ centile at 4 months and the 5^th^ centile at 8 months based upon the normative data have been shown to be predictive of abnormal development at 18 months based on a sample of 201 infants at risk of adverse motor development.[23] Whilst the AIMS is not appropriate to evaluate the longitudinal changes in development of infants with CP,[21] the purpose of the current study is to examine the accuracy of the AIMS in detecting later motor problems, including CP.

#### Neuro-Sensory Motor Development Assessment (NSMDA)

The NSMDA was designed in Australia to assess development over a period of time, grading qualitatively and quantitatively the same aspects of neurosensory-motor development from the ages 1 month to 6 years.[24] Items are administered to evaluate six domains of development including gross motor, fine motor, neurological status, infant patterns of movement, postural development, and motor responses to sensory input. The NSMDA is criterion-referenced and has no normative data; rather the infants are given a classification of motor performance as normal, minimal, mild, moderate or severe dysfunction based on functional grades for each of the six domains. Classification on the NSMDA at 8 months, 2 years and 4 years has been shown to correlate with later motor impairment in children without CP at 11–13 years of age.[25]

### Outcome Measures

#### Movement Assessment Battery for Children, 2^nd^ Edition (MABC-2)

At 4 years’ corrected age, motor outcome was assessed using the MABC-2 at the Royal Children’s Hospital by one of two physiotherapists who were blinded to the child’s previous assessment results and clinical history. No formal training is required, however, reliability between assessors was established prior to study commencement. The MABC-2 includes three subscales: manual dexterity, aiming and catching, and balance, which are summed to give a total motor score.[26] The MABC-2 is reliable and valid in assessing motor development of children from 3 to 16 years of age.[27] Raw scores are converted to percentile ranks using normative data from a sample from the United Kingdom, with scores <15^th^ centile used to classify the child “at-risk of motor difficulty” and <5^th^ centile “significant motor difficulty”.

#### Cerebral Palsy and Gross Motor Function Classification System

A diagnosis of CP was made by the child’s paediatrician and confirmed by the assessing physiotherapist at 4 years. The gross motor function classification system (GMFCS) was used to further classify motor function for children with CP.[28]

### Statistical analysis

All data were analysed using Stata version 13. Summary statistics (means and SD for continuous and numbers and percentage for categorical variables) were used to compare perinatal characteristics between children who did and did not participate in the 4-year assessment, restricted to those in the substudy of serial motor assessments. Children’s motor development was classified as delayed if they scored <10^th^ centile at 4 months and <5^th^ centile at 8 and 12 months on the AIMS, and if the child was categorised in the mild to severe motor dysfunction range on the NSMDA. Sensitivity, specificity, positive and negative predictive values, accuracy, and the positive and negative likelihood ratios, along with 95% confidence intervals (CIs), were used to assess the AIMS and NSMDA at 4, 8 and 12 months as a diagnostic tool for predicting MABC-2 scores at ≤5^th^ and ≤15^th^ centile and CP at 4 years. This analysis was repeated using the number of delayed infant assessments (i.e. 1 or more delayed, 2 or more delayed, or all 3 delayed) on the AIMS and NSMDA, and whether they were delayed on both assessment tools at a given time point (i.e. delayed on both) to examine the predictive validity of multiple assessment time points and the combination of the two assessments.

## Results

**Fig 1:**
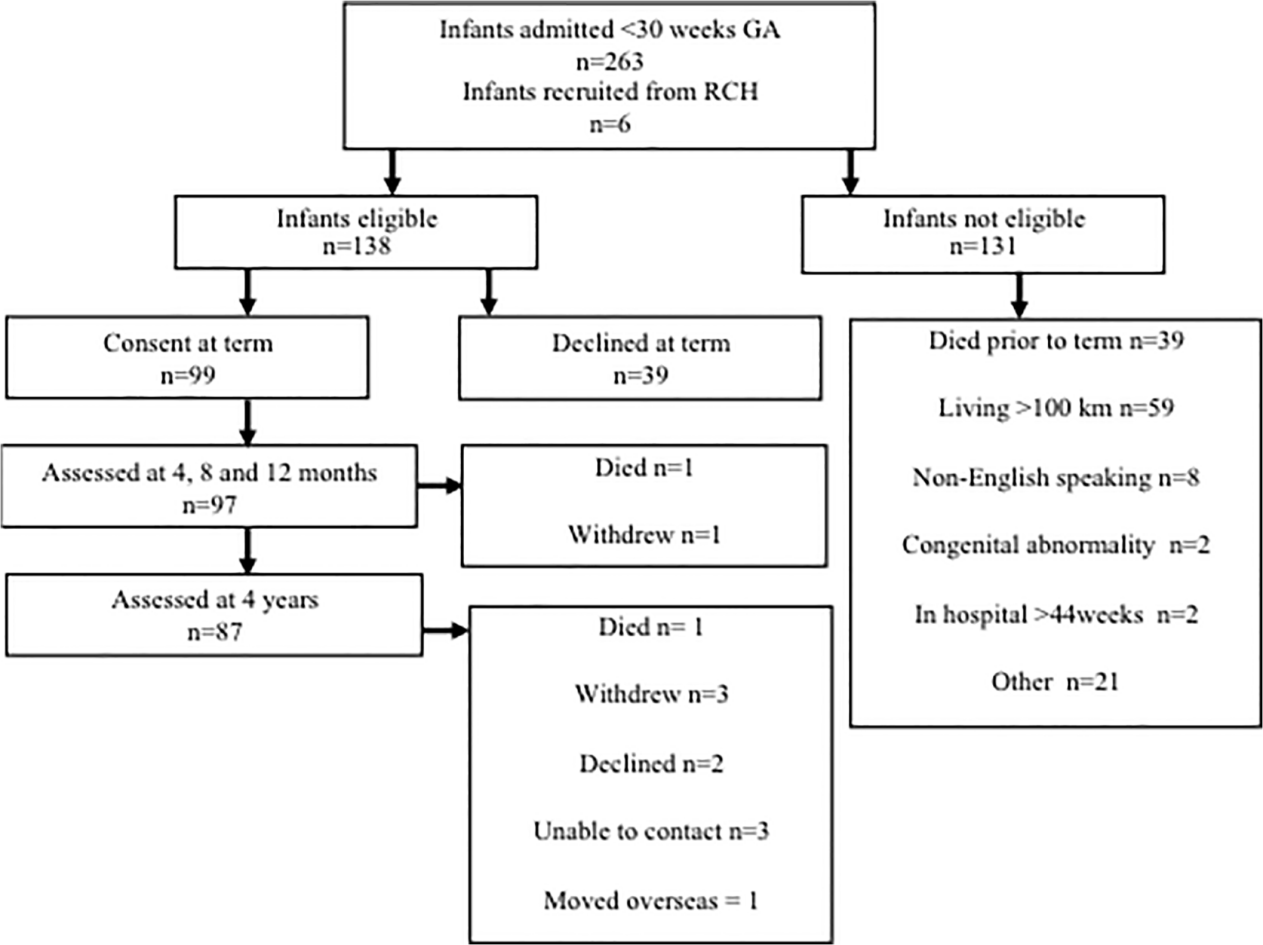
Flow chart of recruitment and follow-up assessments.

Of the 99 infants, two died (one at 4 months and one at 2 years) and 10 did not return for follow-up, resulting in 87 children (88% of survivors) being assessed for CP at 4 years (Fig 1). Of the 87 children, 5 children did not complete the full MABC-2, resulting in 82 children being assessed for motor outcome on the MABC-2. The perinatal characteristics and rates of motor delay were similar between children who did and did not return for follow-up (Table 1). At 4 years, six children had CP (GMFCS, three were level II, two level III and one level V) and all scored <5^th^ centile on the MABC-2. Overall, 26% of the children scored ≤15^th^ centile on the MABC-2. Of the 6 children with CP, 2 had no intraventricular haemorrhage (IVH), 1 had grade I IVH, 1 grade III IVH and 2 had grade IV. Of the remaining 5 children who had grade III/IV, 1 scored ≤5^th^ centile on the MABC-2, 1 scored between 5^th^-15^th^ centile on the MABC-2 and 3 scored within the normal range.

**Table 1:** Characteristics of the study sample.

Perinatal characteristics		Followed-up at 4 years (n = 87)	Not follow-up at 4 years (n = 10)*
Birth weight (g)—mean (SD)		1027 (29)	918 (84)
Gestational age (weeks)—mean (SD)		27.3 (1.2)	27.1 (1.5)
Male gender—n (%)		47 (54)	3 (30)
Multiple birth—n (%)		26 (30)	2 (30)
Postnatal corticosteroids—n (%)		5 (6)	0 (0)
Grade III/IV intraventricular haemorrhage—n (%)		8 (9)	0 (0)
Oxygen at 36 weeks—n (%)		30 (34)	2 (20)
**Motor outcome—n (%)**			
4 months	AIMS <10^th^ centile	19 (22)	3 (30)
	NSMDA mild-severe	18 (21)	2 (20)
8 months	AIMS <5^th^ centile	23 (26)	3 (30)
	NSMDA mild-severe	24 (28)	2 (20)
12 months	AIMS <5^th^ centile	31 (36)	4 (40)
	NSMDA mild-severe	16 (18)	2 (20)
4 years	MABC-2* <5^th^ Centile	21 (26)	-
	MABC-2* <15^th^ Centile	18 (22)	-
	Cerebral Palsy	6 (7)	-

Note: Two infants died prior to 2 years and are not included in this table; AIMS = Alberta Infant Motor Scale; NSMDA = Neuro-Sensory Motor Developmental Assessment; MABC-2 = Movement Assessments Battery for Children 2^nd^ edition

*n = 82; SD = standard deviation

The sensitivity, specificity, positive and negative predictive value, accuracy and positive and negative likelihood rations of the AIMS and NSMDA assessments for predicting motor outcomes at 4 years are reported in Table 2. For the MABC-2, the AIMS had relatively low sensitivity at all time-points with better specificity at 4 and 8 months than at 12 months. The NSMDA also had low sensitivity for the MABC-2 but high specificity at all three time points. The AIMS and NSMDA had 100% sensitivity at 8 and 12 months for CP, but one CP case was not identified using either scale at 4 months.

**Table 2:** Sensitivity, specificity, positive and negative predictive values, positive and negative likelihood ratios and accuracy for the Alberta Infant Motor Scale and the Neuro-Sensory Motor Developmental Assessment at 4, 8 and 12 months for predicting motor impairment at 4 years.

Age	Delayed on the predictor n (%)	Motor outcome at 4 years	Impaired on the outcome n (%)	Sensitivity (95%CI)	Specificity (95%CI)	+ve PV (95%CI)	-ve PV (95%CI)	LR+ (95% CI)	LR- (95% CI)	Accuracy (95% CI)
**Alberta Infant Motor Scale**										
4 months	19 (22%)	MABC<15^th^	21 (26%)	52 (30, 74)	89 (78, 95)	61 (36, 83)	84 (73, 92)	4.6 (2.0, 10.2)	0.5 (0.3, 0.9)	**79 (69, 87)**
		MABC<5^th^	18 (22%)	61 (36, 83)	89 (79, 95)	61 (36, 83)	89 (79, 95)	5.6 (2.5, 12.3)	0.4 (0.2, 0.8)	**83 (73, 90)**
		CP	6 (7%)	83 (36, 100)	83 (73, 90)	26 (9, 51)	99 (92, 100)	4.8 (2.7, 8.7)	0.2 (0.0, 1.2)	83 (73, 90)
8 months	23 (26%)	MABC<15^th^	21 (26%)	57 (34, 78)	85 (74, 93)	57 (34, 78)	85 (74, 93)	3.9 (1.9, 7.9)	0.5 (0.3, 0.8)	78 (68, 86)
		MABC<5^th^	18 (22%)	61 (36, 83)	84 (73, 92)	52 (30, 74)	89 (78, 95)	3.9 (2.0, 7.7)	0.5 (0.3, 0.8)	79 (69, 87)
		CP	6 (7%)	100 (54, 100)	79 (69, 87)	26 (10, 48)	100 (94, 100)	4.8 (3.1, 7.3)	*	80 (71, 88)
12 months	31 (36%)	MABC<15^th^	21 (26%)	71 (48, 89)	75 (63, 86)	50 (31, 69)	88 (77, 96)	2.9 (1.7, 4.9)	0.4 (0.2, 0.8)	74 (64, 83)
		MABC<5^th^	18 (22%)	78 (52, 94)	75 (63, 85)	47 (28, 66)	92 (81, 98)	3.1 (1.9, 5.1)	0.3 (0.1, 0.7)	76 (65, 84)
		CP	6 (7%)	100 (54, 100)	69 (58, 79)	19 (7, 37)	100 (94, 100)	3.2 (2.3, 4.5)	*	71 (61, 80)
**Neuro-Sensory Motor Developmental Assessment**										
4 months	18 (21%)	MABC<15^th^	21 (26%)	38 (18, 62)	85 (74, 93)	47 (23, 72)	80 (68, 89)	2.6 (1.1, 5.8)	0.7 (0.5, 1.0)	73 (62, 82)
		MABC<5^th^	18 (22%)	44 (22, 69)	86 (75, 93)	47 (23, 72)	85 (74, 92)	3.2 (1.4, 7.0)	0.6 (0.4, 1.0)	77 (66, 85)
		CP	6 (7%)	83 (36, 100)	84 (74, 91)	28 (10, 53)	99 (92, 100)	5.2 (2.8, 9.6)	0.2 (0.0, 1.2)	84 (74, 91)
8 months	24 (28%)	MABC<15^th^	21 (26%)	52 (30, 74)	82 (70, 91)	50 (28, 72)	83 (71, 92)	2.9 (1.5, 5.7)	0.6 (0.4, 0.9)	74 (64, 83)
		MABC<5^th^	18 (22%)	56 (31, 78)	81 (70, 90)	45 (24, 68)	87 (75, 94)	3.0 (1.5, 5.7)	0.5 (0.3, 0.9)	76 (65, 84)
		CP	6 (7%)	100 (54, 100)	78 (67, 86)	25 (10, 47)	100 (94, 100)	4.5 (3.0, 6.8)	*	79 (69, 87)
12 months	16 (18%)	MABC<15^th^	21 (26%)	38 (18, 62)	90 (80, 96)	57 (29, 82)	81 (70, 89)	3.9 (1.5, 9.9)	0.7 (0.5, 1.0)	77 (66, 85)
		MABC<5^th^	18 (22%)	39 (17, 64)	89 (79, 95)	50 (23, 77)	84 (73, 92)	3.6 (1.4, 8.8)	0.7 (0.5, 1.0)	78 (68, 86)
		CP	6 (7%)	100 (54, 100)	88 (78, 94)	38 (15, 65)	100 (95, 100)	8.1 (4.5, 14.5)	*	**89 (80, 94**)
**Age**	**Delayed on the predictor n (%)**	**Motor outcome at 4 years**	**Impaired on the outcome n (%)**	**Sensitivity (95%CI)**	**Specificity (95%CI)**	**+ve PV (95%CI)**	**-ve PV (95%CI)**	**+ve LR (95% CI)**	**-ve LR (95% CI)**	**Accuracy (95% CI)**
**Alberta Infant Motor Scale**										
4 months	19 (22%)	MABC<15^th^ MABC<5^th^ CP	21 (26%)	52 (30, 74)	89 (78, 95)	61 (36, 83)	84 (73, 92)	4.6 (2.0, 10.2)	0.5 (0.3, 0.9)	**79 (69, 87)**
			18 (22%)	61 (36, 83)	89 (79, 95)	61 (36, 83)	89 (79, 95)	5.6 (2.5, 12.3)	0.4 (0.2, 0.8)	**83 (73, 90)**
			6 (7%)	83 (36, 100)	83 (73, 90)	26 (9, 51)	99 (92, 100)	4.8 (2.7, 8.7)	0.2 (0.0, 1.2)	83 (73, 90)
8 months	23 (26%)	MABC<15^th^ MABC<5^th^ CP	21 (26%)	57 (34, 78)	85 (74, 93)	57 (34, 78)	85 (74, 93)	3.9 (1.9, 7.9)	0.5 (0.3, 0.8)	78 (68, 86)
			18 (22%)	61 (36, 83)	84 (73, 92)	52 (30, 74)	89 (78, 95)	3.9 (2.0, 7.7)	0.5 (0.3, 0.8)	79 (69, 87)
			6 (7%)	100 (54, 100)	79 (69, 87)	26 (10, 48)	100 (94, 100)	4.8 (3.1, 7.3)	*	80 (71, 88)
12 months	31 (36%)	MABC<15^th^ MABC<5^th^ CP	21 (26%)	71 (48, 89)	75 (63, 86)	50 (31, 69)	88 (77, 96)	2.9 (1.7, 4.9)	0.4 (0.2, 0.8)	74 (64, 83)
			18 (22%)	78 (52, 94)	75 (63, 85)	47 (28, 66)	92 (81, 98)	3.1 (1.9, 5.1)	0.3 (0.1, 0.7)	76 (65, 84)
			6 (7%)	100 (54, 100)	69 (58, 79)	19 (7, 37)	100 (94, 100)	3.2 (2.3, 4.5)	*	71 (61, 80)
**Neuro-Sensory Motor Developmental Assessment**										
4 months	18 (21%)	MABC<15^th^ MABC<5^th^ CP	21 (26%)	38 (18, 62)	85 (74, 93)	47 (23, 72)	80 (68, 89)	2.6 (1.1, 5.8)	0.7 (0.5, 1.0)	73 (62, 82)
			18 (22%)	44 (22, 69)	86 (75, 93)	47 (23, 72)	85 (74, 92)	3.2 (1.4, 7.0)	0.6 (0.4, 1.0)	77 (66, 85)
			6 (7%)	83 (36, 100)	84 (74, 91)	28 (10, 53)	99 (92, 100)	5.2 (2.8, 9.6)	0.2 (0.0, 1.2)	84 (74, 91)
8 months	24 (28%)	MABC<15^th^ MABC<5^th^ CP	21 (26%)	52 (30, 74)	82 (70, 91)	50 (28, 72)	83 (71, 92)	2.9 (1.5, 5.7)	0.6 (0.4, 0.9)	74 (64, 83)
			18 (22%)	56 (31, 78)	81 (70, 90)	45 (24, 68)	87 (75, 94)	3.0 (1.5, 5.7)	0.5 (0.3, 0.9)	76 (65, 84)
			6 (7%)	100 (54, 100)	78 (67, 86)	25 (10, 47)	100 (94, 100)	4.5 (3.0, 6.8)	*	79 (69, 87)
12 months	16 (18%)	MABC<15^th^ MABC<5^th^ CP	21 (26%)	38 (18, 62)	90 (80, 96)	57 (29, 82)	81 (70, 89)	3.9 (1.5, 9.9)	0.7 (0.5, 1.0)	77 (66, 85)
			18 (22%)	39 (17, 64)	89 (79, 95)	50 (23, 77)	84 (73, 92)	3.6 (1.4, 8.8)	0.7 (0.5, 1.0)	78 (68, 86)
			6 (7%)	100 (54, 100)	88 (78, 94)	38 (15, 65)	100 (95, 100)	8.1 (4.5, 14.5)	*	**89 (80, 94**)

Note-CI = Confidence Interval, CP = Cerebral Palsy, MABC = Movement Assessment Battery for Children-2^nd^ edition; PV = Predictive Value; LR = Likelihood Ratio; Results are all percentages except for LR; Numbers in bold reflect the most accurate assessment for each outcomes

* Cannot calculate the LR- when the—ve PV is 100%

In regards to serial motor assessments using the AIMS, 48 (60%) children consistently performed within the normal range, whilst 17 (21%) had one delayed assessment, 10 (12%) had two and 12 (15%) had three. For the NSMDA, 59 (72%) children were consistently classified within the normal-minimal motor dysfunction range, 7 (9%) had one delayed assessment, 12 (15%) had two and 9 (11%) had three. The accuracy was best when all 3 time points demonstrated delay rather than just a single assessment, with the positive likelihood ratio for motor impairment increasing with the number of delayed assessments (Table 3).

**Table 3:** Sensitivity, specificity, positive and negative predictive values and likelihood ratios for the number of delayed assessments on the Alberta Infant Motor Scale and the Neuro-Sensory Motor Developmental Assessment at 4, 18 and 12 months for predicting motor impairment at 4 years.

Delayed assessments	Delayed on the predictor n (%)	Motor outcome at 4 years	Impaired on the outcome n(%)	Sensitivity (95%CI)	Specificity (95%CI)	+ve PV (95%CI)	-ve PV (95%CI)	LR+ (95% CI)	LR- (95% CI)	Accuracy (95% CI)
**Alberta Infant Motor Scale**										
1 or more	39 (45%)	MABC<15^th^	21 (26%)	81 (58, 95)	67 (54, 79)	46 (29, 63)	91 (79, 98)	2.5 (1.6, 3.7)	0.3 (0.1, 0.7)	71 (60, 80)
		MABC<5^th^	18 (22%)	89 (65, 99)	67 (54, 78)	43 (27, 61)	96 (85, 99)	2.7 (1.8, 4.0)	0.2 (0.0, 0.6)	72 (61, 81)
		CP	6 (7%)	100 (54, 100)	59 (48, 70)	15 (6, 31)	100 (93, 100)	2.5 (1.9, 3.2)	*	62 (51, 72)
2 or more	22 (25%)	MABC<15^th^	21 (26%)	57 (34, 78)	87 (76, 94)	60 (36, 81)	85 (74, 93)	4.4 (2.1, 9.2)	0.5 (0.3, 0.8)	79 (69, 87)
		MABC<5^th^	18 (22%)	61 (36, 83)	86 (75, 93)	55 (32, 77)	89 (78, 95)	4.3 (2.1, 8.8)	0.5 (0.3, 0.8)	80 (70, 88)
		CP								
			6 (7%)	100 (54, 100)	80 (70, 88)	27 (11, 50)	100 (94, 100)	5.1 (3.3, 7.9)	*	82 (72, 89)
All 3	12 (14%)	MABC<15^th^	21 (26%)	43 (22, 66)	95 (86, 99)	75 (43, 95)	83 (72, 91)	8.7 (2.6, 29.2)	0.6 (0.4, 0.9)	**82 (72, 89)**
		MABC<5^th^	18 (22%)	50 (26, 74)	95 (87, 99)	75 (43, 95)	87 (77, 94)	10.7 (3.2, 35.3)	0.5 (0.3, 0.8)	**85 (76, 92)**
		CP								
			6 (7%)	83 (36, 100)	91 (83, 96)	42 (15, 72)	99 (93, 100)	9.6 (4.4, 21.3)	0.2 (0.0, 1.1)	91 (83, 96)
**Neuro-Sensory Motor Developmental Assessment**										
1 or more	28 (32%)	MABC<15^th^	21 (26%)	52 (30, 74)	75 (63, 86)	42 (23, 63)	82 (70, 91)	2.1 (1.2, 3.9)	0.6 (0.4, 1.0)	70 (58, 79)
		MABC<5^th^	18 (22%)	56 (31, 78)	75 (63, 85)	38 (20, 59)	86 (74, 94)	2.2 (1.2, 4.0)	0.6 (0.3, 1.0)	71 (60, 80)
		CP								
			6 (7%)	100 (54, 100)	73 (62, 82)	21 (8, 41)	100 (94, 100)	3.7 (2.6, 5.3)	*	75 (64, 83)
2 or more	21 (24%)	MABC<15^th^	21 (26%)	52 (30, 74)	87 (76, 94)	58 (33, 80)	84 (73, 92)	4.0 (1.9, 8.6)	0.5 (0.3, 0.9)	78 (68, 86)
		MABC<5^th^	18 (22%)	56 (31, 78)	86 (75, 93)	53 (29, 76)	87 (77, 94)	4.0 (1.9, 8.2)	0.5 (0.3, 0.9)	79 (69, 87)
		CP	6 (7%)	100 (54, 100)	81 (71, 89)	29 (11, 52)	100 (95, 100)	5.4 (3.4, 8.5)	*	83 (73, 90)
All 3	9 (10%)	MABC<15^th^	21 (26%)	24 (8, 47)	95 (86, 99)	63 (24, 91)	78 (67, 87)	4.8 (1.3, 18.5)	0.8 (0.6, 1.0)	77 (66, 85)
		MABC<5^th^	18 (22%)	28 (10, 53)	95 (87, 99)	63 (24, 91)	82 (72, 90)	5.9 (1.6, 22.5)	0.8 (0.6, 1.0)	80 (70, 88)
		CP	6 (7%)	83 (36, 100)	95 (88, 99)	56 (21, 86)	99 (93, 100)	16.9 (6.1, 46.8)	0.2 (0.0, 1.0)	**94 (87, 98)**
**Delayed assessments**	**Delayed on the predictor n (%)**	**Motor outcome at 4 years**	**Impaired on the outcome (n)**	**Sensitivity (95%CI)**	**Specificity (95%CI)**	**+ve PV (95%CI)**	**-ve PV (95%CI)**	**+ve LR (95% CI)**	**-veLR (95% CI)**	**Accuracy (95% CI)**
**Alberta Infant Motor Scale**										
1 or more	39 (45%)	CP MABC<15^th^ MABC<5^th^	21 (26%)	81 (58, 95)	67 (54, 79)	46 (29, 63)	91 (79, 98)	2.5 (1.6, 3.7)	0.3 (0.1, 0.7)	71 (60, 80)
			18 (22%)	89 (65, 99)	67 (54, 78)	43 (27, 61)	96 (85, 99)	2.7 (1.8, 4.0)	0.2 (0.0, 0.6)	72 (61, 81)
			6 (7%)	100 (54, 100)	59 (48, 70)	15 (6, 31)	100 (93, 100)	2.5 (1.9, 3.2)	*	62 (51, 72)
2 or more	22 (25%)	CP MABC<15^th^ MABC<5^th^	21 (26%)	57 (34, 78)	87 (76, 94)	60 (36, 81)	85 (74, 93)	4.4 (2.1, 9.2)	0.5 (0.3, 0.8)	79 (69, 87)
			18 (22%)	61 (36, 83)	86 (75, 93)	55 (32, 77)	89 (78, 95)	4.3 (2.1, 8.8)	0.5 (0.3, 0.8)	80 (70, 88)
			6 (7%)	100 (54, 100)	80 (70, 88)	27 (11, 50)	100 (94, 100)	5.1 (3.3, 7.9)	*	82 (72, 89)
All 3	12 (14%)	CP MABC<15^th^ MABC<5^th^	21 (26%)	43 (22, 66)	95 (86, 99)	75 (43, 95)	83 (72, 91)	8.7 (2.6, 29.2)	0.6 (0.4, 0.9)	**82 (72, 89)**
			18 (22%)	50 (26, 74)	95 (87, 99)	75 (43, 95)	87 (77, 94)	10.7 (3.2, 35.3)	0.5 (0.3, 0.8)	**85 (76, 92)**
			6 (7%)	83 (36, 100)	91 (83, 96)	42 (15, 72)	99 (93, 100)	9.6 (4.4, 21.3)	0.2 (0.0, 1.1)	91 (83, 96)
**Neuro-Sensory Motor Developmental Assessment**										
1 or more	28 (32%)	CP MABC<15^th^ MABC<5^th^	21 (26%)	52 (30, 74)	75 (63, 86)	42 (23, 63)	82 (70, 91)	2.1 (1.2, 3.9)	0.6 (0.4, 1.0)	70 (58, 79)
			18 (22%)	56 (31, 78)	75 (63, 85)	38 (20, 59)	86 (74, 94)	2.2 (1.2, 4.0)	0.6 (0.3, 1.0)	71 (60, 80)
			6 (7%)	100 (54, 100)	73 (62, 82)	21 (8, 41)	100 (94, 100)	3.7 (2.6, 5.3)	*	75 (64, 83)
2 or more	21 (24%)	CP MABC<15^th^ MABC<5^th^	21 (26%)	52 (30, 74)	87 (76, 94)	58 (33, 80)	84 (73, 92)	4.0 (1.9, 8.6)	0.5 (0.3, 0.9)	78 (68, 86)
			18 (22%)	56 (31, 78)	86 (75, 93)	53 (29, 76)	87 (77, 94)	4.0 (1.9, 8.2)	0.5 (0.3, 0.9)	79 (69, 87)
			6 (7%)	100 (54, 100)	81 (71, 89)	29 (11, 52)	100 (95, 100)	5.4 (3.4, 8.5)	*	83 (73, 90)
All 3	9 (10%)	CP MABC<15^th^ MABC<5^th^	21 (26%)	24 (8, 47)	95 (86, 99)	63 (24, 91)	78 (67, 87)	4.8 (1.3, 18.5)	0.8 (0.6, 1.0)	77 (66, 85)
			18 (22%)	28 (10, 53)	95 (87, 99)	63 (24, 91)	82 (72, 90)	5.9 (1.6, 22.5)	0.8 (0.6, 1.0)	80 (70, 88)
			6 (7%)	83 (36, 100)	95 (88, 99)	56 (21, 86)	99 (93, 100)	16.9 (6.1, 46.8)	0.2 (0.0, 1.0)	**94 (87, 98)**

Note-CI = Confidence Interval, CP = Cerebral Palsy, MABC = Movement Assessment Battery for Children-2^nd^ edition; PV = Predictive Value; LR = Likelihood Ratio; Results are all percentages except for LR; Numbers in bold reflect the most accurate assessment for each outcomes

* Cannot calculate the—ve LR when the—ve PV is 100%

When combining both motor tools, only 13% were delayed at 4 months on both tools, 22% at 8 months and 16% at 12 months. Four months had the best accuracy for all types of motor impairment, although the results were similar at 8 and 12 months (Table 4). Of those with CP, all were delayed on both the NSMDA and AIMS at 8 and 12 months.

**Table 4:** Sensitivity, specificity, positive and negative predictive values and likelihood ratios for being impaired on both the Alberta Infant Motor Scale and the Neuro-Sensory Motor Developmental Assessment at 4, 18 and 12 months for predicting motor impairment at 4 years.

Age at assessment	Delayed on the predictor n (%)	Motor outcome at 4 years	Impaired on the outcome n (%)	Sensitivity (95%CI)	Specificity (95%CI)	+ve PV (95%CI)	-ve PV (95%CI)	LR+ (95% CI)	LR- (95% CI)	Accuracy % (95% CI)
4 months	11 (13%)	MABC<15^th^	21 (26%)	38 (18, 62)	95 (86, 99)	73 (39, 94)	82 (71, 90)	7.7 (2.3, 26.5)	0.7 (0.5, 0.9)	**80 (70, 88)**
		MABC<5^th^	18 (22%)	44 (22, 69)	95 (87, 99)	73 (39, 94)	86 (76, 93)	9.5 (2.8, 32.1)	0.6 (0.4, 0.9)	**84 (74, 91)**
		CP	6 (7%)	83 (36, 100)	93 (85, 97)	45 (17, 77)	99 (93, 100)	11.3 (4.8, 26.3)	0.2 (0.0, 1.1)	**92 (84, 97)**
8 months	19 (22%)	MABC<15^th^	21 (26%)	52 (30, 74)	89 (78, 95)	61 (36, 83)	84 (73, 92)	4.6 (2.0, 10.2)	0.5 (0.3, 0.9)	79 (69, 87)
		MABC<5^th^	18 (22%)	56 (31, 78)	88 (77, 94)	56 (31, 78)	88 (77, 94)	4.4 (2.1, 9.6)	0.5 (0.3, 0.9)	80 (70, 88)
		CP	6 (7%)	100 (54, 100)	84 (74, 91)	32 (13, 57)	100 (95, 100)	6.2 (3.8, 10.3)	*	85 (76, 92)
12 months	14 (16%)	MABC<15^th^	21 (26%)	38 (18, 62)	92 (82, 97)	62 (32, 86)	81 (70, 90)	4.6 (1.7, 12.6)	0.7 (0.5, 1.0)	78 (68, 86)
		MABC<5^th^	18 (22%)	39 (17, 64)	91 (81, 96)	54 (25, 81)	84 (73, 92)	4.1 (1.6, 10.8)	0.7 (0.5, 1.0)	79 (69, 87)
		CP	6 (7%)	100 (54, 100)	90 (81, 96)	43 (18, 71)	100 (95, 100)	10.1 (5.2, 19.5)	*	91 (83, 96)

Note-CI = Confidence Interval, CP = Cerebral Palsy, MABC = Movement Assessment Battery for Children-2^nd^ edition; PV = Predictive Value; LR = Likelihood Ratio; Results are all percentages except for LR; Numbers in bold reflect the most accurate assessment for each outcomes

* Cannot calculate the LR- when the—ve PV is 100%

## Discussion

This is the first prospective study to report serial motor assessment of very preterm children using the AIMS and NSMDA during the first year of life for predicting long-term motor outcome at 4 years. The rate of motor delay in infancy differed according to the assessment tool used. The rate of children falling below the cut-offs progressively increased with age during the first year on the AIMS, whilst it peaked at 8 months with the NSMDA. When using a single time point to assess later motor outcome, delay on the MABC-2 at age 4 was most accurately predicted by the AIMS at 4 months, and CP at age 4 by the NSMDA at 12 months. Prediction of motor impairment at age 4 years was improved when results from all 3 assessment time points were utilised. Accuracy was further improved when results from both assessments at each time point were combined.

The benefit of serial, longitudinal assessment of motor development in infancy to assist in more accurate prediction and diagnosis of later motor impairment in very preterm children is supported by the current study findings.[17, 22] Whilst the AIMS and NSMDA at 4, 8 and 12 months were predictive of later motor impairment, there were several infants who performed within the normal range at an early assessment who fell below the cut-off at a later assessment during the first year, including one infant who was later diagnosed with CP. Similarly, many infants who were initially classified with motor delay went on to have a normal motor outcome at 4 years. Barbosa et al had comparable findings in a study of 10 infants diagnosed with CP who were assessed serially over the first year of life on the AIMS at 3, 6, 9 and 12 months.[29] In their study the number of infants with CP classified as delayed in infancy increased with age, however, unlike our study there was no age where all infants with CP were identified as delayed. Burns et al investigated the predictive value of the NSMDA at 1, 4 and 8 months in a cohort of 26 infants who were later diagnosed with CP. The results showed that prediction improved with time, with over-identification of infants at 4 months, similar to the current study.[30] Whilst our study supports the use of motor assessments at 4 months’ corrected age in preterm infants for predicting later impairment, our findings highlight the importance of reassessing performance at a later age. Further, infants who are at high-risk of later motor impairments but perform within the normal range, should not be discharged from follow-up at 4 months of age after a normal assessment as motor problems might become evident with time.

The specific assessment tool used to identify motor delay in the first year influences prediction, with the AIMS classifying a larger number of infants with delay at one or more time points than the NSMDA. This may be explained by the AIMS only assessing gross motor development through observation, whilst the NSMDA involves handling the infant to assess a range of motor areas.[13] In addition, the AIMS compares an individual infant’s score to normative data, whilst the NSMDA is criterion referenced. There are other differences between the AIMS and NSMDA that are important to consider in interpreting the results of our study. The AIMS is easy to administer due to the observational nature of the assessment and can be carried out by any health professional. On the other hand, the NSMDA is designed for use by physiotherapists and occupational therapists with expertise in infant handling and development, which may limit its clinical utility in some settings. Although the AIMS can be used from 1 to 18 months, it has been reported to have floor and ceiling effects when used prior to 3 months and later than 12 months in infants born preterm.[7,31,32] In line with this, we found the lowest accuracy of the AIMS at 12 months, and we recommend caution when using the AIMS at this age. The NSMDA is a more traditional neuromotor assessment and may provide more detailed diagnostic information, such as increased muscle tone or asymmetries. The disadvantage is that some infants might resist being handled by the assessor. The NSMDA has not been used as widely in clinical practice and research as has the AIMS, with only a small number of studies assessing its predictive validity.[13, 33] Using the assessment tools in combination across the three infant time-points during infancy can provide complementary information if the goal is to monitor for a range of motor impairments as the AIMS is better for predicting motor impairment on the MABC-2, whilst the NSMDA is better for predicting CP. However, if the goal of the assessment is to detect CP, the NSMDA is recommended.

Prediction of motor impairment is challenging due to the multifactorial elements involved in child development, and in both clinical and research settings we are unlikely to be able to predict with absolutely certainty whether a child will go on to have cerebral palsy or another developmental impairment from one assessment alone.[34] It is important to consider the infant’s whole clinical history when reporting assessment results to families. Further, when examining the predictive value of an assessment tool there is often a trade-off between sensitivity and specificity. For example, when predicting motor impairment on the MABC-2, as specificity scores increased with the number of assessments used, the sensitivity decreased. If the goal of follow-up assessments is to determine which children are at-risk and require early intervention, higher sensitivity is preferred. However, higher specificity is preferred if the goal is to ensure that services are not directed to those with no impairment. In most situations a balance between sensitivity and specificity is recommended.

The strengths of the current study include the use of two standardised motor assessments to monitor development of the same cohort of very preterm children over time with high follow-up rates. However, there are some limitations. There were 5 children who were unable to complete the MABC-2 at 4 years due to behaviour issues, which may result in underestimation of motor impairment at 4 years. Long term motor outcome was assessed at 4 years, an important time to assess motor function because children are reaching school age, however the validity of assessments at this age has been questioned.[25] As preterm children grow older, motor assessments tend to identify more problems, most likely due to the complexity of assessment tasks increasing with age.[25,33,35,36] It will be important to assess the motor functioning of these children at older ages, as the diagnosis of motor impairment might change. Additionally, whilst this study has focused on the predictive validity of two infant motor assessments, in evidence-based clinical practice it is important to use a holistic approach to assessment, including family and medical history and results from neuro-imaging when available.[34]

In conclusion, assessment of infants during the first year of life is an important part of neonatal follow-up programs to examine and predict motor development, and to ensure intervention is targeted to those at greatest risk.[37] The AIMS and NSMDA used at 4, 8 and 12 months’ corrected age are predictive of later motor outcome, including CP and motor impairment assessed with the MABC-2 at 4 years.
